# MEG Source Imaging and Group Analysis Using VBMEG

**DOI:** 10.3389/fnins.2019.00241

**Published:** 2019-03-22

**Authors:** Yusuke Takeda, Keita Suzuki, Mitsuo Kawato, Okito Yamashita

**Affiliations:** ^1^ATR Neural Information Analysis Laboratories, Kyoto, Japan; ^2^ATR Brain Information Communication Research Laboratory Group, Kyoto, Japan

**Keywords:** VBMEG, MEG, EEG, fMRI, source imaging, source reconstruction, Bayes

## Abstract

Variational Bayesian Multimodal EncephaloGraphy (VBMEG) is a MATLAB toolbox that estimates distributed source currents from magnetoencephalography (MEG)/electroencephalography (EEG) data by integrating functional MRI (fMRI) (https://vbmeg.atr.jp/). VBMEG also estimates whole-brain connectome dynamics using anatomical connectivity derived from a diffusion MRI (dMRI). In this paper, we introduce the VBMEG toolbox and demonstrate its usefulness. By collaborating with VBMEG's tutorial page (https://vbmeg.atr.jp/docs/v2/static/vbmeg2_tutorial_neuromag.html), we show its full pipeline using an open dataset recorded by Wakeman and Henson (2015). We import the MEG data and preprocess them to estimate the source currents. From the estimated source currents, we perform a group analysis and examine the differences of current amplitudes between conditions by controlling the false discovery rate (FDR), which yields results consistent with previous studies. We highlight VBMEG's characteristics by comparing these results with those obtained by other source imaging methods: weighted minimum norm estimate (wMNE), dynamic statistical parametric mapping (dSPM), and linearly constrained minimum variance (LCMV) beamformer. We also estimate source currents from the EEG data and the whole-brain connectome dynamics from the MEG data and dMRI. The observed results indicate the reliability, characteristics, and usefulness of VBMEG.

## 1. Introduction

Both magnetoencephalography (MEG) and electroencephalography (EEG) measure electrical neural activities and have excellent temporal resolution on the millisecond order. However, estimating source currents from them is an ill-posed problem because the number of sensors is insufficient to precisely reconstruct the source currents. We cannot identify from them a unique source current that only generates MEG/EEG data. To solve this problem, prior information about the source current is necessary to reduce the solution space. Several prior assumptions have been used, such as the minimum norm method (Hämäläinen et al., 1993; Hämäläinen and Ilmoniemi, 1994) and the maximum smoothness method (Pascual-Marqui et al., 1994). However, their prior assumptions are insufficient to reconstruct the source current with high spatial resolution. An alternative is to obtain prior information from other modalities, such as functional MRI (fMRI), which measures hemodynamic responses to neural activities. Although it has low temporal resolution owing to slow hemodynamic responses, it has high spatial resolution on the millimeter order. Therefore, using fMRI activity as prior information provides a source current with high spatiotemporal resolution. Generally, integrating multimodal measurements effectively alleviates the ill-posed nature of MEG/EEG source imaging and provides reliable and informative knowledge of human brain activities.

Variational Bayesian Multimodal EncephaloGraphy (VBMEG), which is a Matlab toolbox, estimates distributed source currents and connectome dynamics from MEG and/or EEG data by integrating such multimodal measurements as fMRI. VBMEG was originally developed to perform a hierarchical Bayesian source current estimation proposed by Sato et al. (2004), and the first version was released in 2011 (https://vbmeg.atr.jp/). Its reliability was confirmed in various studies by our group (Yoshioka et al., 2008; Callan et al., 2010; Aihara et al., 2012; Takeda et al., 2014) and others (Toda et al., 2011; Yoshimura et al., 2012, 2017; Yamagishi and Anderson, 2013; Morioka et al., 2014; Callan et al., 2016; Ohata et al., 2016; Yanagisawa et al., 2016; Fukuma et al., 2018; Mejia et al., 2018; Sato et al., 2018). Recently, VBMEG was extended to perform a connectome dynamics estimation proposed by Fukushima et al. (2015), and its second version was released in 2017. Its usefulness was also confirmed by Filatova et al. (2018).

VBMEG's main advantage is its ability to integrate multimodal measurements for improving estimation accuracies. In estimating source currents, VBMEG can integrate fMRI activity for improved source localization accuracy (Sato et al., 2004). Dynamic statistical parametric mapping (dSPM) can also integrate fMRI activity (Liu et al., 1998; Dale et al., 2000). This method uses fMRI activity as prior information on the source current variance. In contrast, VBMEG uses fMRI activity as prior information on the variance distribution rather than the variance itself to produce a soft constraint on the variance. Because of this, VBMEG is also robust to inaccurate fMRI activities (Sato et al., 2004; Aihara et al., 2012). In estimating connectome dynamics, VBMEG uses anatomical connectivity derived from a diffusion MRI (dMRI). Without assuming region of interests (ROIs), it estimates a whole-brain linear dynamics model by only assuming connectivity coefficients between anatomically connected regions. This drastically reduces the connectivity coefficients to estimate and suppress false positive connectivities (Filatova et al., 2018).

Although VBMEG's algorithms (Sato et al., 2004; Fukushima et al., 2015) and their application results have been published, VBMEG itself has not been introduced yet. In this paper, we introduce the VBMEG toolbox and demonstrate its usefulness. In collaboration with VBMEG's tutorial page (https://vbmeg.atr.jp/docs/v2/static/vbmeg2_tutorial_neuromag.html), we show its full pipeline using an open dataset recorded by Wakeman and Henson (2015). We import the MEG data and preprocess them to estimate source currents. From the estimated source currents, we perform a group analysis and examine the differences of current amplitudes between conditions by controlling the false discovery rate (FDR), which yields results consistent with previous studies. To highlight VBMEG's characteristics, we compared these results with those obtained by other source imaging methods: weighted minimum norm estimate (wMNE), dSPM (Liu et al., 1998; Dale et al., 2000) and linearly constrained minimum variance (LCMV) beamformer (Van Veen et al., 1997). We also estimate the source currents from the EEG data and the whole-brain connectome dynamics from the MEG data and dMRI. The observed results indicate the reliability, characteristics, and usefulness of VBMEG.

## 2. General Information

### 2.1. VBMEG's Aim

**Figure 1:**
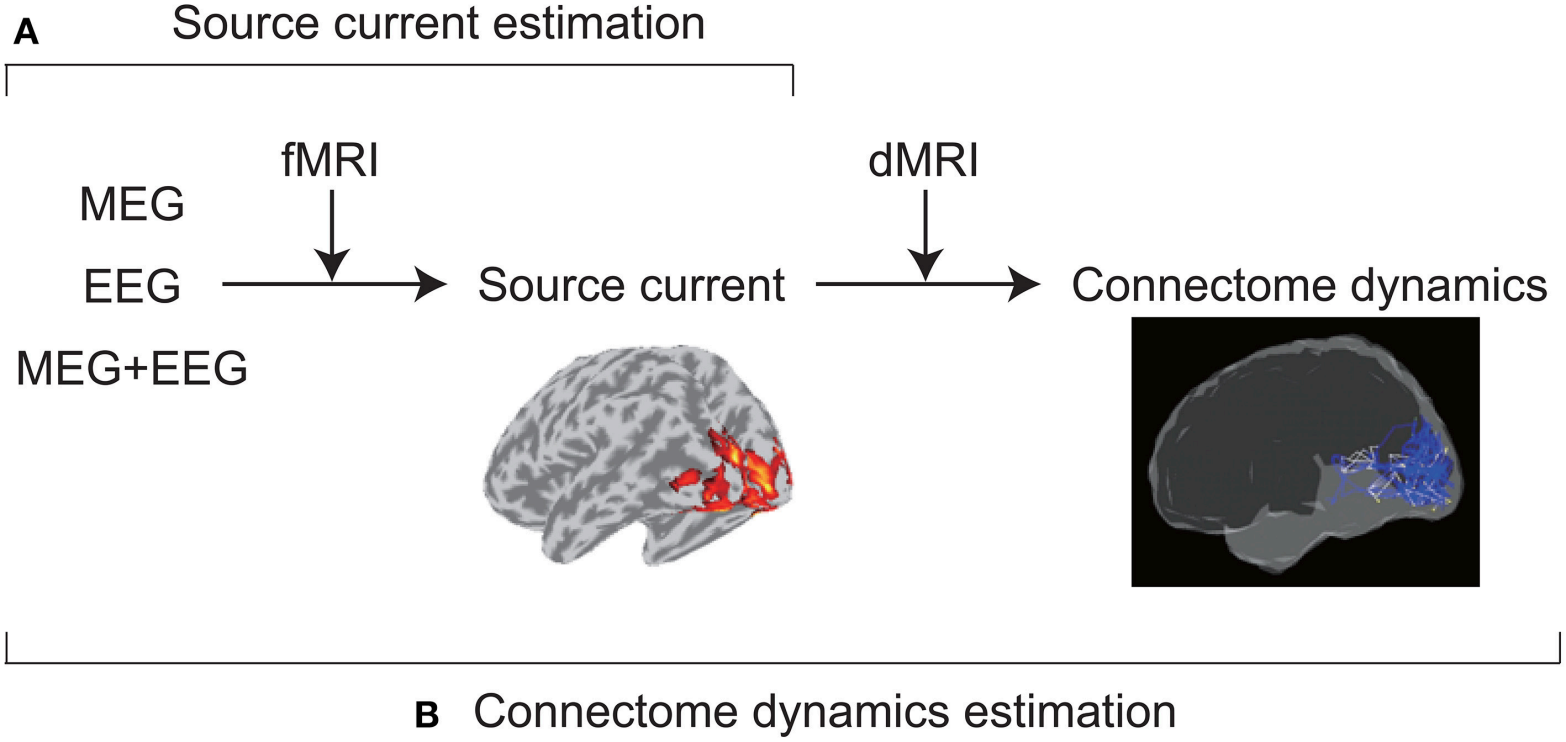
Two main VBMEG functions. **(A)** From MEG and/or EEG data, VBMEG estimates source currents by integrating fMRI activity. **(B)** It also estimates whole-brain connectome dynamics by integrating anatomical connectivity derived from a dMRI.

VBMEG was developed to achieve accurate source imaging by integrating multimodal measurements (Figure 1). From MEG and/or EEG data, VBMEG estimates source currents using fMRI activity as prior information on current variance distribution (Sato et al., 2004). VBMEG also estimates whole-brain connectome dynamics using anatomical connectivity derived from a dMRI (Fukushima et al., 2015). The estimated dynamics are visualized by a movie that displays signal flows (https://vbmeg.atr.jp/gallery/ for example movies).

### 2.2. Starting VBMEG

To start VBMEG, go to its web page (https://vbmeg.atr.jp/), which provides an introduction and a download link.

Several VBMEG usages can also be learned through tutorials (https://vbmeg.atr.jp/document/). Using actual experimental data, they describe step-by-step procedures from importing raw data to visualizing the estimation results of source currents and connectome dynamics using a graphical user interface (GUI) or batch scripts.

### 2.3. System Requirements

#### 2.3.1. Operating System

A GNU Linux is strongly recommended because VBMEG is usually developed and tested on Linux. Its use on Microsoft Windows 7/10 and Apple OS X hasn't been satisfactorily tested yet.

#### 2.3.2. Software

VBMEG works on MATLAB (version 7 [R14] to 8.3 [R2014a]). Signal Processing Toolbox is needed to process MEG/EEG data. FreeSurfer4.2 or newer (http://surfer.nmr.mgh.harvard.edu/) is needed to extract cortical surfaces from T1 images. SPM8 (https://www.fil.ion.ucl.ac.uk/spm/software/spm8/) is also needed to process the T1 images and fMRIs. For estimating connectome dynamics, MRtrix 0.2.1x (https://www.nitrc.org/projects/mrtrix/) and FSL 4.1 or newer (http://www.fmrib.ox.ac.uk/fsl) are also needed for processing dMRIs.

## 3. Tutorial

By collaborating with VBMEG's tutorial page, we introduce its pipeline. We import the open MEG dataset recorded by Wakeman and Henson (2015) and preprocess it to estimate the source currents. For each subject, we assume 10,004 current dipoles perpendicular to the cortical surface and estimate their currents by integrating the fMRI activity. Then from the estimated source currents of all the subjects, we examine the differences of the current amplitudes between conditions. Furthermore, we estimate the source currents from the EEG data and the whole-brain connectome dynamics from the MEG data and dMRI.

This tutorial was developed using MATLAB 2013b with a Signal Processing Toolbox on Linux where MRtrix 0.2.10 and FSL 4.1 had been installed.

### 3.1. Starting Tutorial

We analyze the multi-subject, multi-modal neuroimaging dataset for face processing (OpenNEURO ds000117-v1.0.1) created by Wakeman and Henson (2015). This dataset contains the evoked responses of 16 subjects to three types of face stimuli: famous, unfamiliar, and scrambled. MEG, EEG, electro-oculograms (EOGs), and electro-cardiograms (ECGs) were simultaneously recorded at 1,100 Hz with an Elekta Neuromag Vectorview 306 system (Helsinki). T1 images and fMRIs were also collected with a Siemens 3T TIM TRIO (Siemens, Erlangen, Germany). These data are stored in the Brain Imaging Data Structure (BIDS) format (http://bids.neuroimaging.io/).

VBMEG defines the current sources on the cortical surfaces based on the FreeSurfer's results, which we prepared in advance because obtaining them is time-consuming. We also prepared the fMRI activities for estimating the current variances by analyzing the fMRI data using SPM8.

To highlight VBMEG's characteristics, we compared its results with those estimated by other source imaging methods: wMNE, dSPM, and LCMV beamformer. They were performed using the functions from the Brainstorm software (Tadel et al., 2011).

This tutorial is started by downloading these data and the software from the following links:

**VBMEG**

      https://vbmeg.atr.jp/download2/

**SPM8**

      https://www.fil.ion.ucl.ac.uk/spm/software/spm8/

**Brainstorm**

      https://neuroimage.usc.edu/brainstorm/

**MEG data (OpenNEURO ds000117-v1.0.1)**

      https://openneuro.org/datasets/ds000117/versions/1.0.1/

**Tutorial programs, FreeSurfer's results, and fMRI activities**

      https://vbmeg.atr.jp/docs/v2/static/vbmeg2_tutorial_neuromag.html

See the above tutorial page for program-level descriptions. This page also presents the resultant figures and movie that serve as Supplementary Material.

### 3.2. Modeling Brain

VBMEG defines the current sources on each subject's cortical surface, the boundary between the gray and white matter, and stores them as a brain model.

To construct a brain model, we first import the T1 image (.nii) by converting its coordinate system to that of VBMEG, where the orientation is RAS and the origin is the center of the image. The coordinates of the fiducials (left and right pulmonary arteries [LPA and RPA] and the nasion) are also converted from voxels to the VBMEG coordinate system. Because current sources are defined in this coordinate system, the following analyses (including the SPM analysis of the fMRI data) need to be conducted using the imported T1 image.

**Figure 2:**
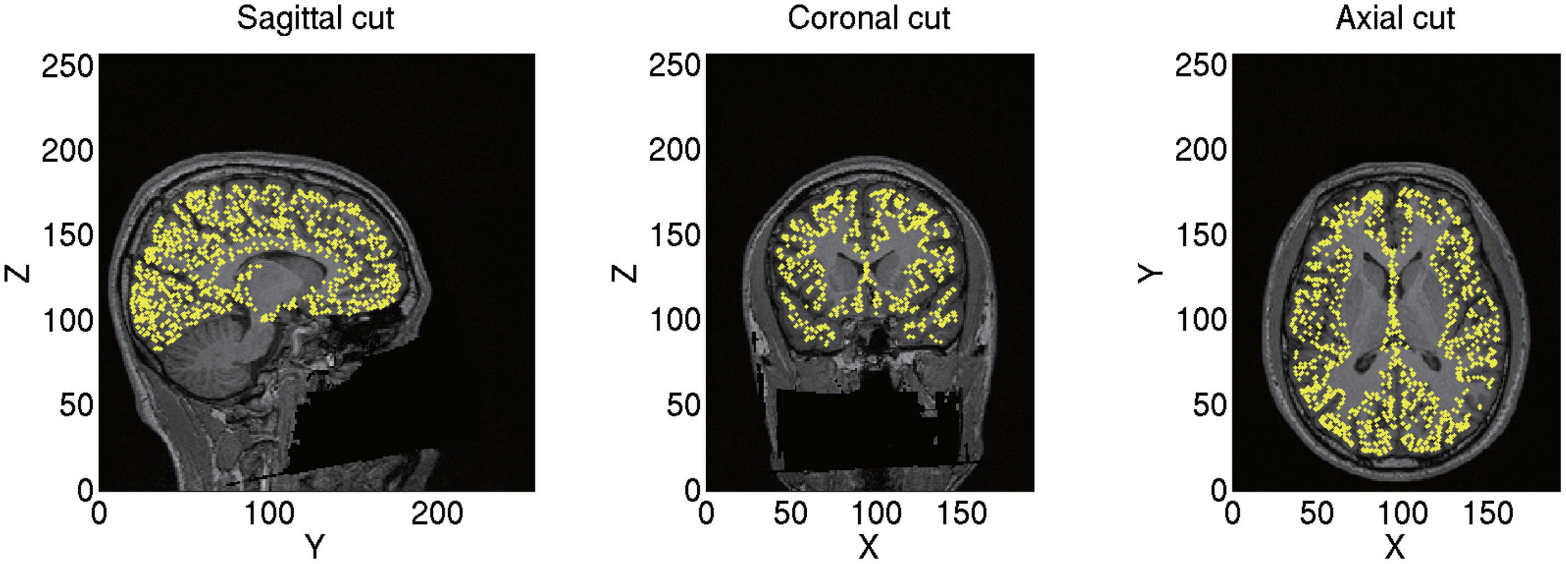
Constructed brain model (sub-08). Current sources are plotted on T1 image by yellow dots.

From the imported T1 image, we construct a polygon model of the cortical surface using FreeSurfer. From the constructed polygon model, we select 10,004 vertices as the current sources based on the predefined sources in a standard brain (MNI-ICBM152). As a result, the sources of different subjects correspond to the same location on the standard brain. For example, the 7975th source is always located at the right fusiform face area (FFA) corresponding to *x* = 38, *y* = −62, and *z* = −18 mm in the Montreal Neurological Institute (MNI) coordinate. This allows a simple comparison of the estimated source currents across subjects for each source. Therefore, we can proceed to group analyses on the source currents without any transformation. The positions of the current sources and their normal directions to the cortical surface are stored as a brain model (.brain.mat). A constructed brain model of sub-08 is shown in Figure 2.

### 3.3. Importing fMRI Activity

**Figure 3:**
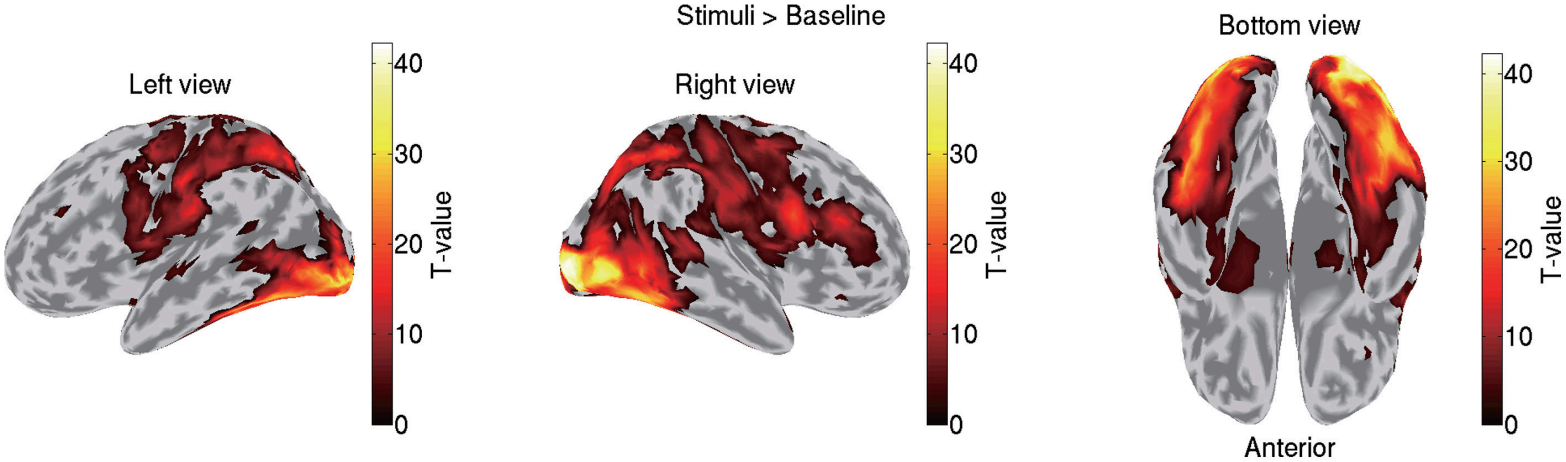
Imported fMRI activity contrasting all stimuli against baseline (sub-08). *T*-values over 0.1 of their maximum value are shown.

VBMEG imports the statistical results of fMRI data generated by SPM8 by mapping voxel *t*-values and percent signal changes to the cortical surface using an inverse-distance weighted interpolation method. We import the statistical results generated by contrasting all the stimuli (famous, unfamiliar, and scrambled) against a baseline. Figure 3 shows the imported fMRI activity of sub-08 plotted on the standard brain. By default, VBMEG plots individual subjects' brain activities on the standard brain.

### 3.4. Preprocessing MEG Data

We preprocess the MEG data for source current estimation.

#### 3.4.1. Importing the MEG Data

VBMEG can import MEG data recorded by Yokogawa and Neuromag systems and EEG data recorded by Biosemi and Brainamp.

**Figure 4:**
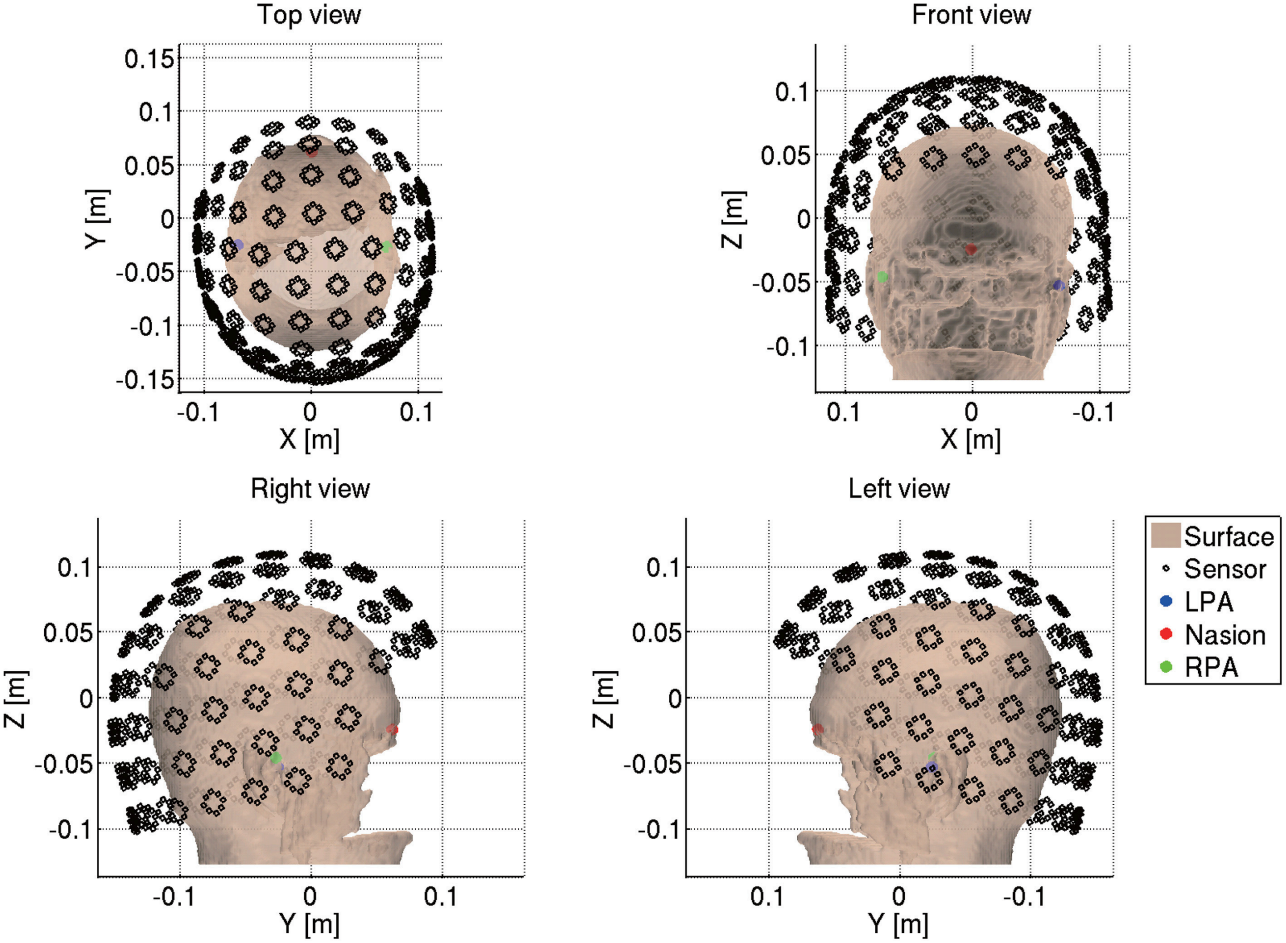
MEG sensor coordinates converted into VBMEG coordinate system (sub-08).

Next we import the Neuromag MEG data (.fif) by reading the .fif files using the functions from the MNE software (http://www.nmr.mgh.harvard.edu/martinos/userInfo/data/MNE_register/index.php) and converting them to the VBMEG format (.meg.mat). We also convert the sensor coordinates to the VBMEG coordinate system by aligning the fiducials and head points recorded in the MEG experiment to the fiducials and the head surface in the VBMEG coordinate system (Figure 4). The head surface is extracted from the T1 image.

After importing the MEG data, we modify the trigger signals (STI101) based on the event files (*_events.tsv). This is because Wakeman and Henson (2015) identified a fixed 34-ms delay between the appearance of a trigger in an MEG file (on channel STI101) and the stimulus's appearance on the screen. We read the stimulus onsets from the event files and make the values of trigger signals 1 and 2 for the face (famous and unfamiliar) and the scrambled conditions 1 s after the stimulus onsets.

#### 3.4.2. Denoising MEG Data

The MEG data include such environmental noises as line and biological noises from eye movements and heartbeats. To remove them, for each channel, we apply a lowpass filter at 40 Hz and a highpass filter at 1 Hz and regress out the EOG and ECG components. We also resample the MEG data at 100 Hz to reduce the computational cost.

#### 3.4.3. Making Trial Data

We detect the stimulus onsets from the trigger signal (STI101) and segment the continuous data into 1.5-s epochs 0.5 s before and 1 s after the stimulus onset.

#### 3.4.4. Combining Trials Across Runs

To handle all the trials collectively, we virtually combine them into one info file (.info.mat). We can load the data of all the trials from the info file using “vb_load_meg_data.m.”

#### 3.4.5. Rejecting Channels and Trials

The info file includes structure array “fileinfo” with “ActiveChannel” and “ActiveTrial” fields. By editing these fields, we can control the channels and the trials to be loaded. We reject the bad channels and the trials by editing them.

**Figure 5:**
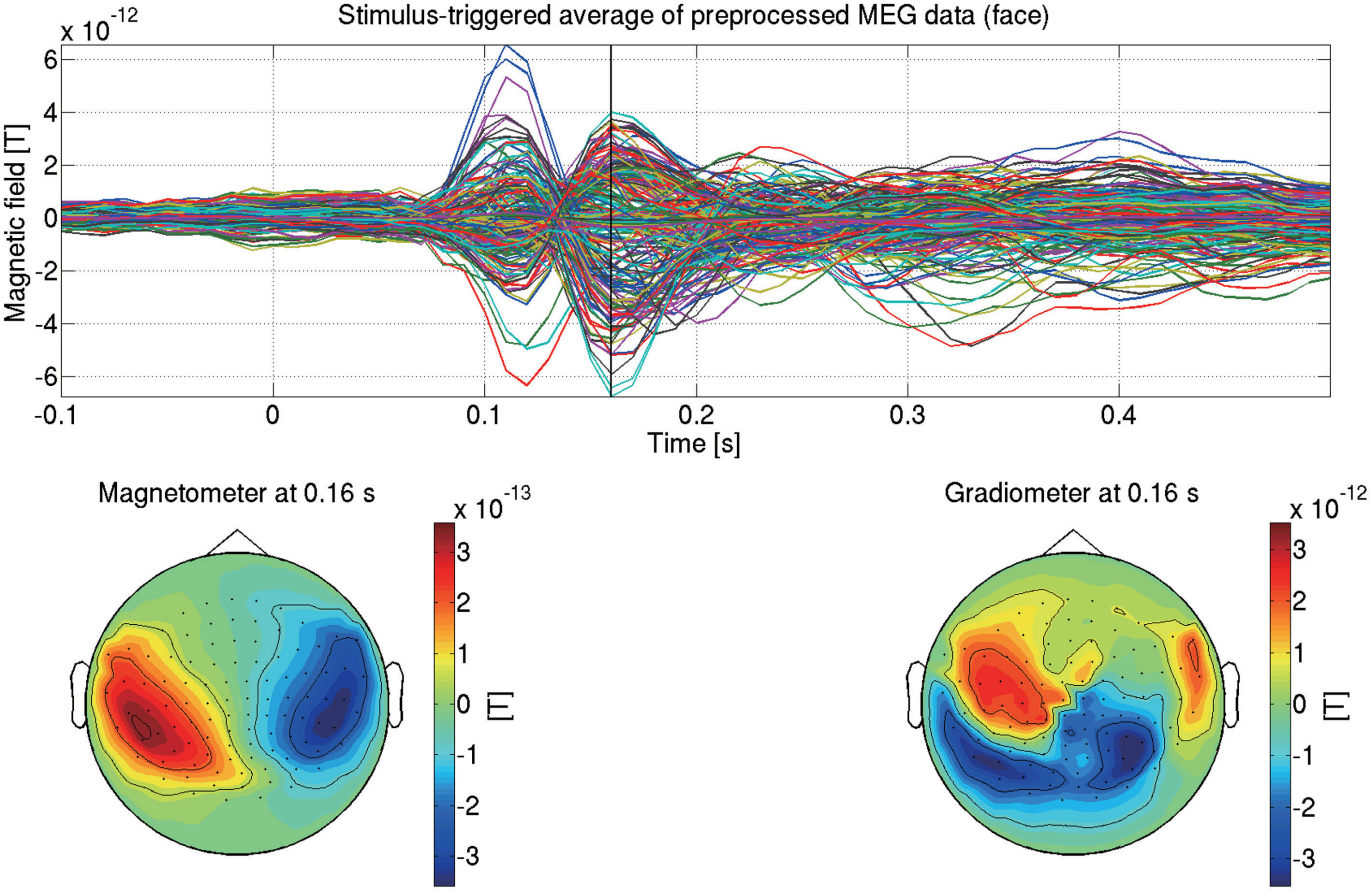
Stimulus-triggered average of preprocessed MEG data in face condition (sub-08). Its time series **(top)** and spatial maps at 0.16 s **(bottom)** are shown.

This completes the preprocessing of the MEG data. Figure 5 shows the preprocessed MEG data of sub-08 in the face condition.

### 3.5. Estimating Source Current From MEG Data

We next estimate the source currents from the preprocessed MEG data.

#### 3.5.1. Preparing Leadfield

VBMEG can respectively construct 1-shell (cerebrospinal fluid [CSF]) and 3-shell (CSF, skull, and scalp) head conductivity models for MEG and EEG source imaging from the cortical surface model obtained by FreeSurfer and the gray matter file obtained by SPM8. Here we construct a 1-shell head conductivity model for MEG source imaging. Based on the model, we make a leadfield matrix by solving the Maxwell equations with a boundary element method (BEM). VBMEG supports three types of dipoles for each source: a one-dimensional dipole perpendicular to the cortical surface, a two-dimensional dipole tangential to the cortical surface, and a three-dimensional dipole parallel to the three axes (x, y, z). Here we assume a one-dimensional dipole.

#### 3.5.2. Estimating the Source Current

In VBMEG, source currents are estimated in two steps: first the current variance and then the source currents.

The current variance is estimated by the hierarchical Bayesian method proposed by Sato et al. (2004). This method is an extension of automatic relevance determination (ARD) (Neal, 1996) in which fMRI activity is incorporated into the hierarchical prior distribution (prior distribution of the relevance parameters). Following the original idea of the sparse promoting nature of ARD, a sparse current is obtained when fMRI activity is unavailable. Indeed, such sparse currents have been estimated by several methods (Matsuura and Okabe, 1995; Uutela et al., 1999; Wipf et al., 2010; Chang et al., 2013; Khan et al., 2014; Bekhti et al., 2018). Here we estimate the current variance by setting a confidence parameter, “bayes_parm.prior_weight,” to 0.3. This parameter controls the confidence in the fMRI prior relative to the amount of data samples (ranging from 0 to 1), where a larger value makes the current variance more closely resemble the fMRI prior.

**Figure 6:**
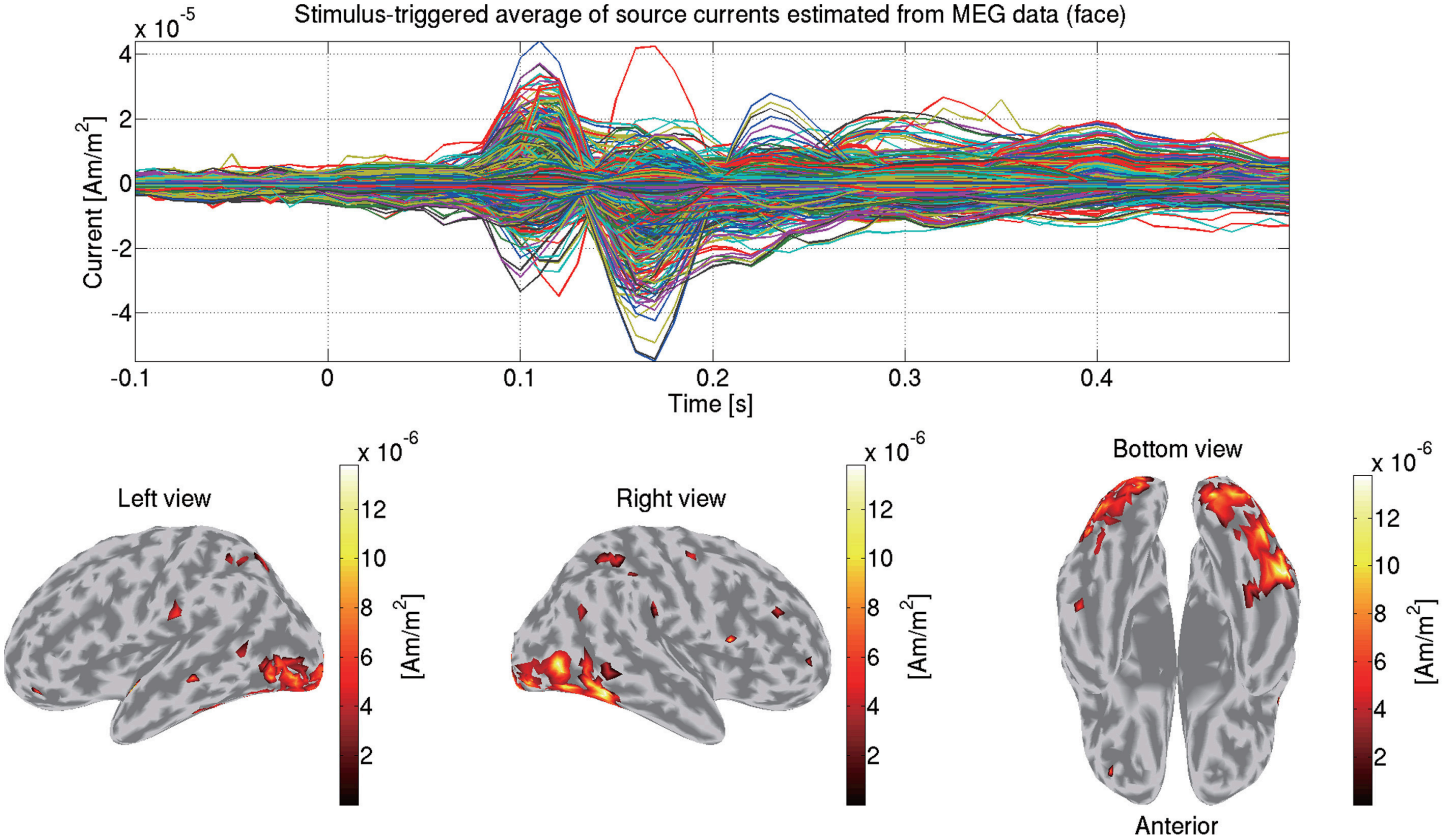
Stimulus-triggered average of source currents estimated from MEG data in face condition (sub-08). Its time series **(top)** and amplitudes averaged within 0–0.3 s **(bottom)** are shown. In bottom figures, activities over 0.3 of their maximum value are shown.

Using the estimated current variance, we make an inverse filter, which is a transformation matrix from the MEG data to the source currents, and estimate the source currents using it. Figure 6 shows the source currents of sub-08 estimated from the MEG data in the face condition.

**Figure 7:**
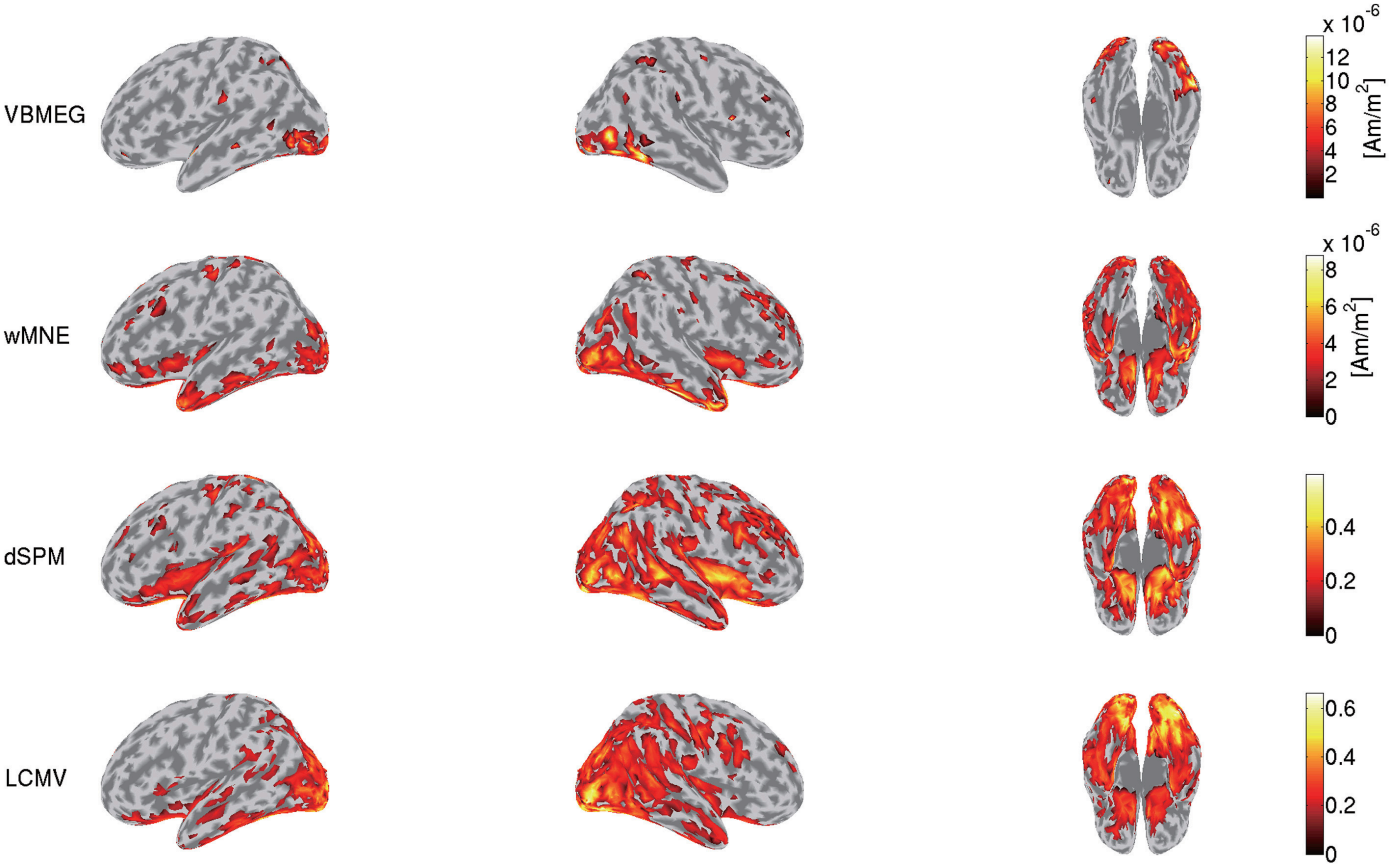
Source currents estimated by VBMEG, wMNE, dSPM, and LCMV beamformer. Stimulus-triggered averages of source currents estimated from MEG data in face condition (sub-08) were calculated, and their amplitudes averaged within 0–0.3 s are shown. For each method, activities over 0.3 of their maximum value are shown.

To highlight VBMEG's characteristics, we compared the source currents estimated by VBMEG with those estimated by other source imaging methods. From the same MEG data and the leadfield matrix used in the above analyses, we estimated the source currents by wMNE, dSPM, and LCMV beamformer using the Brainstorm functions. In applying dSPM, the fMRI activity was not used as prior information on the source current variance because the Brainstorm functions do not support it. Figure 7 shows the source currents estimated by these methods. Compared to wMNE, dSPM, and LCMV beamformer, VBMEG exhibits localized activities around the areas with large fMRI activity (Figures 3, 7).

### 3.6. Group Analyses

Using all the subjects' source currents estimated from the MEG data by VBMEG, we conducted a group analysis and examined the differences of the current amplitudes between the face and scrambled conditions.

For each subject, condition, and source, we calculated the stimulus-triggered average of the source currents estimated from the MEG data, normalized it so that its baseline period (–0.3 to 0 s) has mean 0 and standard deviation 1, and calculated its amplitude. Then for each source and time, we compared the 16 subjects' current amplitudes between the face and scrambled conditions by a paired *t*-test. From the differences of the current amplitudes between the conditions, we calculated the *t*- and *p*-values based on Student's *t*-distribution under a null hypothesis where the current amplitudes were not different between the conditions. This procedure produced a total of 586,515 *p*-values (9,615 sources × 61 time points).

We solved this multiple comparison problem by controlling the FDR, which manages the expected proportion of false positive findings among all the rejected null hypotheses (Benjamini and Hochberg, 1995). We estimated the *q*-values by Storey and Tibshirani's method (2003). From the distribution of the 586,515 *p*-values, we first estimated the proportion of the null *p*-values π_0_, and based on π_0_ we converted the *p*-values to *q*-values. The FDRs were controlled at 0.05. This group analysis was performed by “examine_diff_between_conds.m” in the tutorial programs.

**Figure 8:**
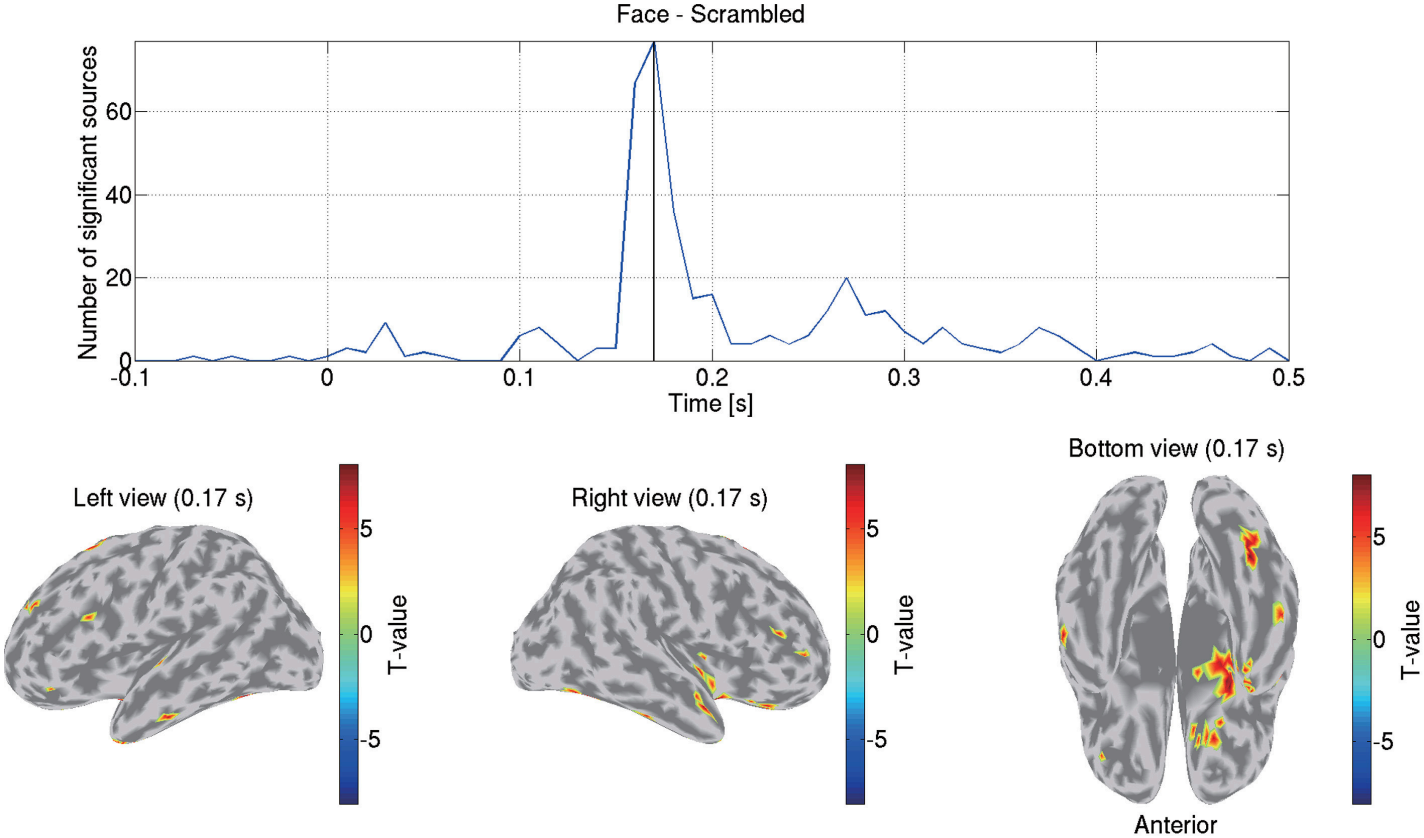
Differences of current amplitudes between face and scrambled conditions. Top figure shows number of sources exhibiting significant differences (*q* < 0.05). Bottom figures show significant *t*-values at 0.17 s.

Figure 8 shows the detected differences between the face and scrambled conditions. At 0.17 s, the largest difference was observed at the right FFA. This result is consistent with previous studies that reported that this area exhibits face-selective responses (Grill-Spector et al., 2004, 2017; Wakeman and Henson, 2015; Jas et al., 2018; Rossion et al., 2018).

**Figure 9:**
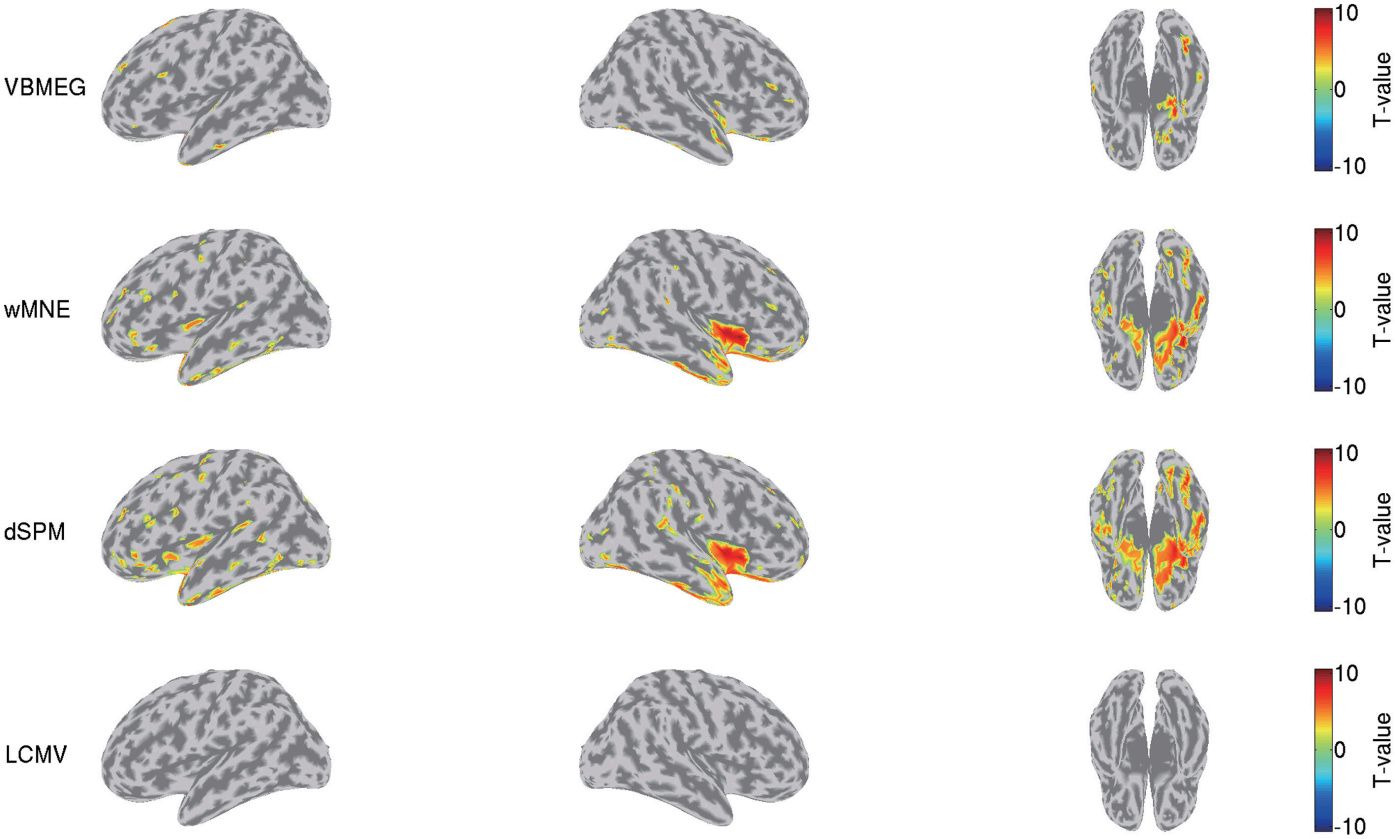
Differences of current amplitudes between face and scrambled conditions obtained by VBMEG, wMNE, dSPM, and LCMV beamformer. Significant *t*-values at 0.17 s are shown (*q* < 0.05).

We also compared this group analysis result with those obtained by wMNE, dSPM, and LCMV beamformer. Using the source currents estimated by these methods (Figure 7), we examined the differences of the current amplitudes between the face and scrambled conditions by the same procedure described above. Figure 9 shows the detected differences between the conditions at 0.17 s. Compared with VBMEG, wMNE and dSPM exhibited significant differences in the broader areas, including the right FFA, the right insula, and the left temporal pole. LCMV beamformer did not exhibit a significant difference at that time.

### 3.7. Estimating Source Current From EEG Data

Here we estimate the source currents from the EEG data.

We import and preprocess them in the same way as the MEG data (section 3.4). Additionally, we take a common average reference and make the averages of the EEG data across the channels to 0.

**Figure 10:**
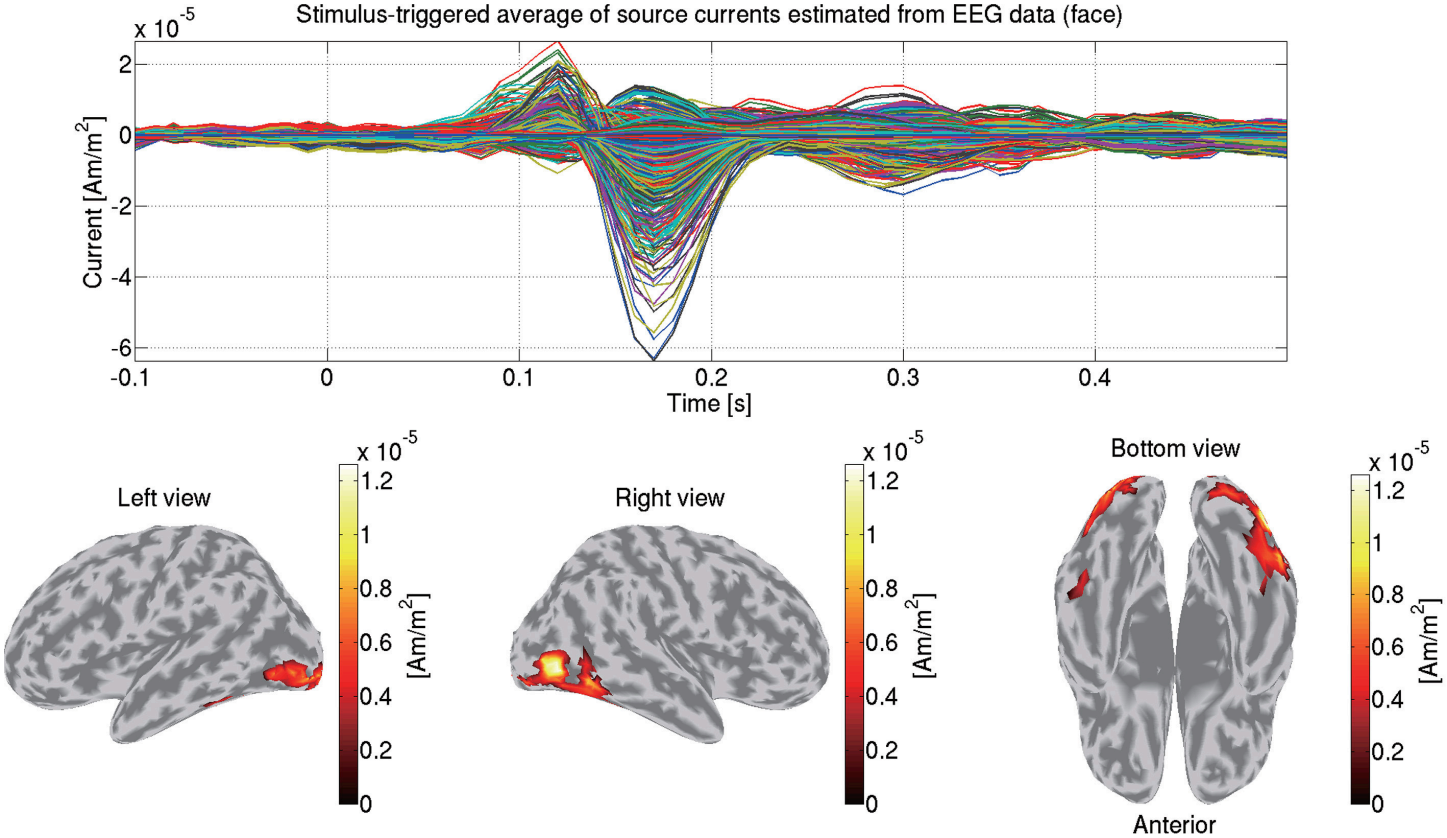
Stimulus-triggered average of source currents estimated from EEG data in face condition (sub-08). Its time series **(top)** and amplitudes averaged within 0–0.3 s **(bottom)** are shown. In bottom figures, activities over 0.3 of their maximum value are shown.

We construct a 3-shell (CSF, skull, and scalp) head conductivity model. The conductivities are respectively set to 0.62, 0.03, and 0.62 S/m for the brain, the skull, and the scalp. Based on the model, we make a leadfield matrix with the common average reference. From the leadfield matrix and the preprocessed EEG data, we estimate the source currents in the same way as the MEG data (section 3.5). Figure 10 shows the source currents of sub-08 estimated from the EEG data in the face condition.

### 3.8. Estimating Source Current From Both MEG and EEG Data

VBMEG can also estimate the source currents from both MEG and EEG data. Because MEG and EEG have different sensitivities to source currents, integrating them further alleviates the ill-posed nature of MEG/EEG source imaging, providing a reliable estimate.

**Figure 11:**
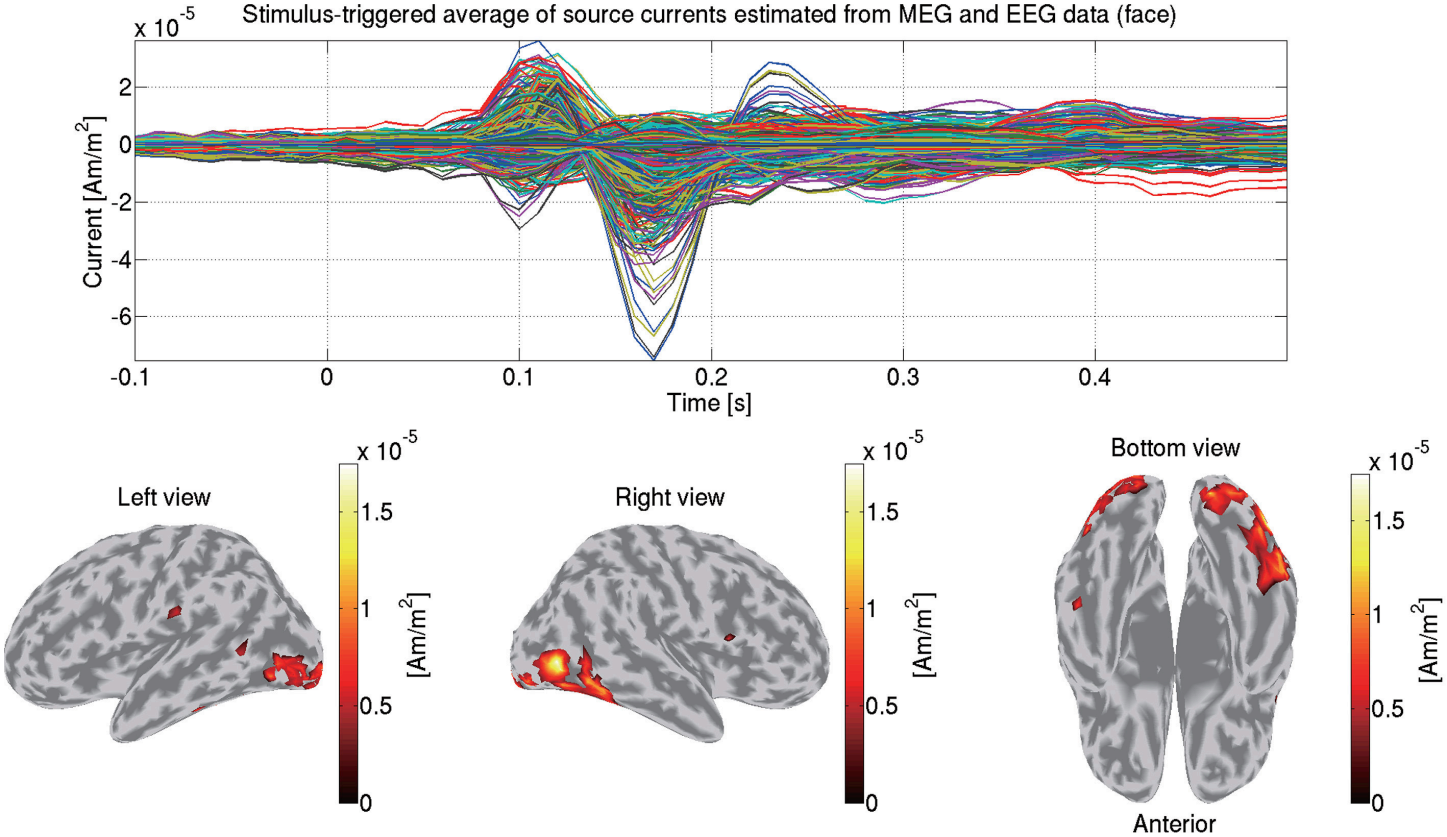
Stimulus-triggered average of source currents estimated from both MEG and EEG data in face condition (sub-08). Its time series **(top)** and amplitudes averaged within 0–0.3 s **(bottom)** are shown. In bottom figures, activities over 0.3 of their maximum value are shown.

We first match the trials between the MEG and EEG data so that identical trials remain. Then from the matched MEG/EEG data and their leadfield matrices, we estimate the source currents. To accommodate the different scales between the MEG and EEG data, the data and leadfield matrices are normalized by the leadfield norms (Henson et al., 2011). The normalized MEG and EEG data and the leadfield matrices are concatenated together. The hierarchical Bayesian method (Sato et al., 2004) is applied to the concatenated data and the leadfield matrices. Figure 11 shows the source currents of sub-08 estimated from both the MEG and EEG data in the face condition.

### 3.9. Estimating Whole-Brain Connectome Dynamics

Finally, we estimate the whole-brain connectome dynamics from the source currents estimated from the MEG data in the face condition. Its procedure consists of two steps: first estimating the anatomical connectivity and then the dynamics model.

The anatomical connectivity is estimated from the dMRI using FSL and MRtrix. We correct the subject motion during the dMRI acquisition by FSL. To obtain ROIs for fiber tracking, we cluster the cortical surfaces into 1,998 parcels. Based on a six-dimensional fiber orientation distribution, the fibers are probabilistically tracked from each ROI using MRtrix. We quantify the strength of the connectivity based on the fiber counts and binarized it using a threshold. The binarized connections are used for specifying pairs of anatomically connected ROIs. Furthermore, from the inter-ROI fiber lengths, we calculate the time lags between the ROIs assuming a fixed conduction velocity at 6 m/s.

Using the anatomical connectivity, we estimate the whole-brain connectome dynamics. We generate the ROI current by averaging the source currents across the trials and the sources within each ROI. From the ROI current, we estimate a linear dynamics model constrained by the anatomical connectivity. In this model, only the anatomically connected pairs of ROIs have connectivity coefficients at the anatomically determined time lags. This drastically reduces the connectivity coefficients to estimate and suppress the false positive connectivities (Filatova et al., 2018). The estimated dynamics model is visualized by a movie showing the signal flows between the ROIs. The created signal flow movie of sub-01 can be seen on the tutorial page.

## 4. Discussion

In this paper, using the open dataset recorded by Wakeman and Henson (2015), we introduced the VBMEG toolbox and demonstrated its practical usage by showing its full pipeline. We imported the MEG data and preprocessed them to estimate the source currents. From the estimated source currents of all the subjects, we performed a group analysis where the face-selective responses were detected by controlling the FDRs. Our results are consistent with previous studies (Grill-Spector et al., 2004, 2017; Wakeman and Henson, 2015; Jas et al., 2018; Rossion et al., 2018), indicating VBMEG's ability to extract reliable knowledge through group analyses.

### 4.1. VBMEG's Advantages

VBMEG's main advantage is its ability to integrate fMRI activity for estimating source currents from MEG/EEG data. Due to this advantage, its estimated source currents tend to be localized around areas with large fMRI activity, and as a result false positive activities are effectively suppressed (Figures 3, 7).

This advantage might also suppress the false positive detection of face-selective responses (Figure 9). So far, face-selective responses have been observed at the ventral occipitotemporal cortex (VOTC) (Grill-Spector et al., 2004, 2017; Wakeman and Henson, 2015; Jas et al., 2018; Rossion et al., 2018). Consistent with these observations, VBMEG, wMNE, and dSPM exhibited face-selective responses at VOTC (Figure 9). However, they also exhibited them outside VOTC, such as insula. This may be due to signal leakage (Brookes et al., 2012; Colclough et al., 2015; Palva et al., 2018; Sato et al., 2018); the face-selective responses at VOTC leaked out in the estimated source currents. Outside VOTC, VBMEG exhibited face-selective responses in narrower areas than wMNE and dSPM (Figure 9), suggesting that integrating the fMRI activity suppressed the signal leakage and the false positive detection.

dSPM can also integrate fMRI activity (Liu et al., 1998; Dale et al., 2000). This method uses fMRI activity as prior information on the current variance, which is computed only from fMRI activity. In contrast, VBMEG uses fMRI activity as prior information on the variance distribution rather than the variance itself, which is computed from both the fMRI activity and the MEG/EEG data. Therefore, VBMEG is also robust to inaccurate fMRI activities (Sato et al., 2004; Aihara et al., 2012).

VBMEG's other advantage is its ability to construct a connectome dynamics model for event-related brain activity. SPM can also construct a dynamics model by dynamic causal modeling (DCM), which constructs a nonlinear dynamics model based on a few predetermined ROIs. In contrast, since VBMEG constructs a linear dynamics model of the whole-brain without assuming such ROIs, it is suitable for revealing whole-brain dynamics in a data-driven way.

### 4.2. VBMEG's Limitations

Currently, VBMEG relies on a few old versions of the software, such as MATLAB (version 7 [R14] to 8.3 [R2014a]) and SPM8. This situation is complicated for young researchers who are interested in trying VBMEG. We plan to extend VBMEG so that it works on more recent versions.

VBMEG supports the importing of MEG data recorded by Yokogawa (.con) and Neuromag (.fif) systems and EEG data recorded by Biosemi (.bdf) and Brainamp (.vhdr, .vmrk, and .eeg). Other formats, such as the European data format (EDF), are currently not supported. We plan to extend the supported formats.

VBMEG does not have a framework to import data processed by other software, such as Brainstorm. To do so, the data must be converted to the VBMEG format. On the other hand, it may be easier to use other software's functions in the VBMEG pipeline. Indeed, VBMEG uses several functions from other free software, such as EEGLAB (Delorme and Makeig, 2004) (https://sccn.ucsd.edu/eeglab/index.php), MRIcron (Rorden et al., 2007) (http://people.cas.sc.edu/rorden/mricron/index.html), and Tools for NIfTI and ANALYZE image (https://jp.mathworks.com/matlabcentral/fileexchange/8797), which are included in the “external/” directory of our toolbox. Furthermore, this tutorial used the Brainstorm functions to estimate the source currents by wMNE, dSPM, and LCMV beamformer.

VBMEG can construct a whole-brain connectome dynamics model. However, since the model is very high-dimensional, it is too complicated to interpret. Further analysis and statistical tests are necessary to extract physiological knowledge from it. They are now under development.

### 4.3. Alternatives to fMRI

VBMEG estimates source currents from MEG/EEG data using fMRI activity as prior information on the current variance distribution. However, measuring fMRIs requires expensive equipment that is not always available. In such cases, we can choose from among several alternatives.

We can use uniform distribution as prior information with very small confidence. In this case, the estimated current variances tend to be sparse due to the effect of ARD (Neal, 1996). Although the estimation accuracy obtained by the uniform prior is worse than that obtained by the fMRI prior, it still outperforms a minimum norm method if the source currents are sparse (Sato et al., 2004).

We can also use near-infrared spectroscopies (NIRSs), which measure hemodynamic responses to neural activities like with fMRIs. NIRS activities mapped to the sources can be used as prior information. The efficacy of this method was validated by Aihara et al. (2012) and Morioka et al. (2014).

On the other hand, obtaining the meta-analysis results of fMRI studies from Neurosynth.org (http://neurosynth.org/) and using them as prior information is another possibility. We are now testing the efficacy of this approach.

### 4.4. Other VBMEG Usages

In this paper, we demonstrated VBMEG usage by analyzing the MEG/EEG data during the face recognition. VBMEG's performances for other experiences have been validated, such as a visual experiment with checkerboard patterns (Yoshioka et al., 2008), a somatosensory experiment with electric stimuli (Filatova et al., 2018), and several motor experiments (Callan et al., 2010, 2016; Toda et al., 2011; Takeda et al., 2014; Yoshimura et al., 2017). Furthermore, its performance can be tested for MEG/EEG data during simple auditory, somatosensory, and visual stimuli through the following tutorial page: https://vbmeg.atr.jp/docs/v2/static/vbmeg2_tutorial_advanced.html.

## Data Availability

All datasets generated for this study are included in the manuscript and/or the supplementary files.

## Author Contributions

YT, MK, and OY designed the work. YT and KS wrote the codes to analyze the data. YT wrote the manuscript. All the authors reviewed the manuscript and approved it for publication.

### Conflict of Interest Statement

The authors declare that the research was conducted in the absence of any commercial or financial relationships that could be construed as a potential conflict of interest.
